# Facilitating Goal-Oriented Behaviour in the Stroop Task: When Executive Control Is Influenced by Automatic Processing

**DOI:** 10.1371/journal.pone.0046994

**Published:** 2012-10-08

**Authors:** Benjamin A. Parris, Sarah Bate, Scott D. Brown, Timothy L. Hodgson

**Affiliations:** 1 Psychology Research Centre, Bournemouth University, Poole, Dorset, UK; 2 School of Psychology, University of Newcastle, Callaghan, New South Wales, Australia; 3 School of Psychology, University of Lincoln, Brayford Pool, Lincoln, UK; Centre national de la recherche scientifique, France

## Abstract

A portion of Stroop interference is thought to arise from a failure to maintain goal-oriented behaviour (or goal neglect). The aim of the present study was to investigate whether goal- relevant primes could enhance goal maintenance and reduce the Stroop interference effect. Here it is shown that primes related to the goal of responding quickly in the Stroop task (e.g. fast, quick, hurry) substantially reduced Stroop interference by reducing reaction times to incongruent trials but increasing reaction times to congruent and neutral trials. No effects of the primes were observed on errors. The effects on incongruent, congruent and neutral trials are explained in terms of the influence of the primes on goal maintenance. The results show that goal priming can facilitate goal-oriented behaviour and indicate that automatic processing can modulate executive control.

## Introduction

The Stroop task requires participants to identify the colour of the font in which a word is presented, whilst ignoring the word itself [1], [2]. When the written word is incongruent with the font colour (e.g. *blue* written in brown), the time it takes to identify the colour is increased relative to a baseline control condition (e.g. *stage* written in brown). This increase in response time is known as Stroop interference. In contrast, when the colour and word are congruent (e.g. *brown* written in brown) the time it takes to identify the colour is decreased relative to the baseline control condition and this decrease is known as Stroop facilitation. These Stroop effects are thought to be the result of an inability to prevent the word dimension from contributing evidence towards a response, leading to competition at the response output level [3], [4].

Whilst the majority of interference is thought be the consequence of response competition, it has been argued that at least a portion of the effects are due to the failure to fully engage the task goal of responding quickly and accurately to the colour dimension of the stimulus [5], [6], [7]. For example, De Jong et al. [6] showed that if the time between the response on trial n-1 and stimulus presentation on trial n (Response-Stimulus Interval: henceforth RSI) was shortened to 200 ms compared to the more common 2000 ms, Stroop interference was not statistically significant. De Jong et al. argued that goal focus (focus on responding quickly and accurately) is improved with a shorter RSI because the task goal is still strongly activated when the next response is needed on the subsequent trial, whereas a longer RSI leads to a neglect of the goal, resulting in larger Stroop effects. They reasoned that instead of the more generally accepted notion that interference results from fundamental limits to inhibitory capacity resulting in response competition, interference might mainly be the result of a failure to fully engage or consistently exploit inhibitory abilities. Efficient performance on any task requires the ability to maintain, monitor and execute a plan of action in line with current goals. Thus, the ability to control and guide novel behaviour is dependent on stable, sustained goal representations.

Duncan and colleagues [8], [9] have shown that goal neglect can be prevented by explicit verbal prompts reminding patients of task goals. The aim of the present study was to investigate whether priming could be used to prompt the goal of responding quickly in the Stroop task thereby reducing interference associated with goal neglect. If primes act as sufficient goal prompts, interference associated with goal neglect could be reduced or even eliminated. This idea was in part inspired by research in social psychology that has shown that priming can lead to the non-conscious activation of goals [10], [11], [12], [13]. Since participants performing the Stroop task already have an activated goal to respond quickly and accurately, the aim of the present experiment was to investigate whether priming can be used to enhance an activated goal that is not fully engaged.

Previous studies have considered the influence of pre-trial cues on the performance of Stroop-like tasks [14], [15], [16], but none has investigated the influence of goal primes. In the present experiment, in order to investigate the influence of goal primes on Stroop task performance, the critical primes were selected by being synonymous with the intentionally activated goal of responding quickly (e.g. fast, hurry). The primes were expected to semantically prime the goal concept of responding quickly thus enabling better response selection. It was predicted that the portion of Stroop interference identified by De Jong et al. [6] and Kane and Engle [7] as being the result of goal neglect would be removed by the primes consistent with the activated goal and that this reduction would be observed as a reduction in the reaction times (RTs) to incongruent trials.

## Methods

### Ethics Statement

Written, informed consent was obtained from all participants prior to taking part in the study. Ethics approval was given by the Ethics Committee in the School of Design, Engineering, and Computing at Bournemouth University.

### Participants

55 proficient English speakers from the University of Bournemouth participated. 7 participants’ data sets were not included in the analysis: 6 due to an awareness of the relevance of the goal primes (see below); 1 due to the reporting of a past traumatic head injury from a car accident some years before. The average age of the remaining 48 subjects was 23.6 years (36 were female).

#### 2.2. Stimuli and materials

The font colours were brown (RGB: 153, 102, 51), green (RGB: 0, 255, 0), white (RGB: 255, 255, 255) and yellow (RGB: 255, 255, 0). The incongruent and congruent stimuli consisted of the colour words *brown, yellow*, *green*, and *white* presented on a black background. The words used for the neutral stimuli were *stage, plenty, plane,* and *large* and were matched for frequency and word length to the colour words above using the MRC psycholinguistic database (http://www.psy.uwa.edu.au/mrcdatabase/uwa_mrc.htm). The critical prime words were *express, fast, hurry, quick, rapid,* and *speedy*. The control prime words were *mill, mixture, send, panel, rural* and *budget*. The two categories of primes were matched for frequency and length using the MRC psycholinguistic database. They were presented in a neutral grey (RGB: 192, 192, 192) and lowercase format. The words were created in Microsoft Powerpoint using Courier New font, size 28, in bold. The visual angles subtended by the words were no smaller than 1.6°×.76° (17 mm long×8 mm high) and no larger than 2.7°×.76° (28 mm long×8 mm high). The stimuli were presented on a PC with a refresh rate of 60 Hz. The experiment was programmed using Experiment Builder (SR Research).

#### 2.3 Procedure

Each participant completed 24 practice Stroop trials. Participants then completed 144 experimental trials. Half of the experimental trials were preceded by critical primes, the other half by the control primes. Thus, each prime condition consisted of 72 trials, 24 of which were incongruent trials, 24 were neutral trials and 24 were congruent trials. Colour combinations were presented equally in the prime type conditions.

At the end of the experiment the participants were asked the following question ‘Do you remember thinking that the words presented in the ‘neutral’ grey were relevant in any way to what you were doing?’ Data from any participant responding ‘Yes’ to this question were not included in the analyses because it was an indication that the participants had paid attention to the primes (only 6 participants’ data were excluded from analysis for this reason).

Each experimental trial began with the presentation of a fixation cross for 200 ms, which was replaced with the prime word which was on the screen for 100 ms. After the offset of the prime, a blank screen was presented for 100 ms after which the Stroop stimulus was presented until participants made a response. A blank screen was then presented for 500 ms before the onset of the fixation cross that began the next trial.

Participants were told to respond as quickly and as accurately as they could to the colour of the font in which the words were presented by pressing one of four keys labelled with colour patches. The keys used were ‘z’, ‘x’, ‘n’ and ‘m’, for white, green, brown and yellow, respectively. They were told that another word would be flashed up in a neutral grey before the onset of the coloured stimulus; which they were also required to ignore.

## Results

### Analysis of Reaction Times

The correct response latencies were subjected to a trimming procedure in which the criterion cut-off for outlier removal is established independently for each participant, for each condition, by reference to the sample size in that condition [17]. Using this procedure, 2.7% of the data were counted as outliers.

The data were entered into a 3 (word type: incongruent/neutral/congruent) ×2 (prime type: goal-consistent/goal-neutral) repeated measures ANOVA. The analysis revealed a main effect of Word type F(2, 94) = 59.246, p<.001, η^2^ = .558, no main effect of Prime type F(1, 47) = .093, p>.7, and a two-way interaction between Word type×Prime type where F(2, 94) = 7.795, p<.01, η^2^ = .142 (See Table 1).

**Table 1:** Reaction times (ms), standard errors (SE) and percentage of errors.

		Goal Primes	Control Primes
Incongruent	RT	719	743
	SE	12	14
	%E	4.3	5.6
Neutral	RT	711	696
	SE	12	12
	%E	4	3.3
Congruent	RT	659	646
	SE	12	11
	%E	3.3	2.8
	Stroop interference	8	47
	Stroop facilitation	52	50

As an important test of the effect, incongruent, neutral and congruent trials were compared across prime conditions. Paired-samples t-tests revealed that reaction times to incongruent trials were significantly reduced by the primes (t(47) = 2.404, p<.05, r = .33), but the reaction times to both the congruent and neutral trials were significantly increased by the primes (t(47) = 2.162, p<.05, r = .3; t(47) = 2.182, p<.05, r = .3, respectively). This pattern of effects led to the substantial reduction of Stroop interference in the goal-consistent prime condition as revealed by paired-samples t-tests (t(47) = 3.244, p<.01, r = .43) but no observed effect on facilitation (t(47) = .098, p>.9). Since this is a key finding for the present study it is important to note that although these are planned comparisons, the finding of reduced Stroop interference would survive the conservative Bonferroni post-hoc correction for familywise error rate as it would require the comparison to be significant at p<.025.

### Analysis of Error Rates

3.9% of responses were recorded as errors. Only the main effect of Word Type (F(2, 94) = 7.193, p<.001, η^2^ = .133) was significant (see Table 1 for percentage errors in each condition).

## Discussion

The aim of the present experiment was to use goal primes to facilitate goal-oriented behaviour in the Stroop task and enhance an already activated, but not fully engaged, goal representation. The results showed that the goal primes significantly reduced Stroop interference by decreasing RTs to incongruent trials and increasing RTs to neutral trials. RTs to congruent trials were also significantly increased, but the concomitant increase in RTs to the neutral trials meant that facilitation was not affected by the primes. These results support the notion that goal neglect might play an important role in determining Stroop effects [6], [7] and that environmental prompts can prevent it [8]. Both in the present study and in the study by De Jong et al. [6] Stroop interference was almost entirely eliminated by experimental manipulations targeting goal neglect, suggesting goal maintenance mechanisms play an important role in selective attention tasks.

An important outcome from the present set of results is that the primes did not speed reaction times to all word types in the goal prime condition relative to the control prime condition. Reaction times were significantly faster to the incongruent stimuli but actually increased for the neutral and congruent stimuli. This suggests that the goal primes are not having a generic speeding effect, or simply increasing phasic alertness, but instead that goal priming affects attention paid to representations in working memory responsible for goal-directed behaviour.

Whilst the decrease in RT to incongruent trials was expected, the increase in RTs to neutral stimuli was not. Although unexpected, one could also account for this increase in terms of facilitated goal maintenance. This possibility is captured in a model of the Stroop task in which activation of the goal to colour name results in inhibition of the pathway responsible for word processing, making the word dimension of the stimulus harder to process [5], [18]. Under this account, facilitating goal maintenance would hinder word processing even further. Consistent with this, research has shown that efficiency of processing can modify colour naming reaction times to neutral trials [19], [20]. For example, Burt [19] showed that colour classification times to low frequency words are slower than those to high frequency words; presumably because low frequency words are harder to process. Conversely, Burt showed that when word recognition processes are facilitated by identity priming (presenting a word as a prime immediately before it appears as the irrelevant dimension of the Stroop stimulus), non-colour word (neutral trial) colour naming times decrease; presumably because they are now easier to process. Likewise, Parris et al. [20] showed that if only one letter of the Stroop stimulus is coloured in one of the response colours in the Stroop task (e.g. the l in blue) and if that letter occupies a position from which processes of visual word recognition are less efficient (away from near the centre of the word; the position known as the Optimal Viewing Position [21]), RTs neutral trials increase, whilst those to incongruent trials decrease. Further support for this comes from Algom, Chajut and Lev [22] who showed that emotion-related words are slower to colour name because they have slower associated word reading times. Taken together, this work indicates that the longer it takes to process neutral words, the longer the associated colour naming RTs, suggesting that word processing has to run to completion before colour naming can occur. Given these findings and the predictions from the model of Cohen and colleagues about increased goal activation and its effect on word processing, the results from the present experiment could be accounted for by assuming that the colour classification goal is facilitated via priming, resulting in hindering the processes of visual word recognition and thus increased colour naming times to neutral stimuli.

The notion that an enhanced colour naming goal hinders word reading also accounts for the increase in RTs to congruent trials. If word reading is hindered then the word dimension of the Stroop stimulus contributes less to the response. When the word contributes less to the response there is less facilitation on congruent trials and thus reaction times increase. However, since Kane and Engle’s [7] work has been part of the motivation for the present work it is important to note that they have argued that as well as a portion of Stroop interference being caused by goal neglect, the entirety of Stroop facilitation results from neglect of the task goal. In contrast to goal neglect that adds to interference (the failure to fully engage goal maintenance mechanisms), they argued that facilitation is the result of a stronger, more serious failure in goal maintenance in which the occasional inadvertent reading of the irrelevant word speeds up the overall RT to congruent trials because reading is faster than colour naming. If this were the case one would have expected that primes that facilitate goal maintenance would also affect the magnitude of facilitation. This was not observed because there was a concomitant increase in neutral and congruent trial RTs in the goal priming condition. Nevertheless, the increase in congruent trials RTs could be taken as being consistent with this position. Importantly, however, Kane and Engle noted that goal neglect need not be an all-or-none phenomenon, and suggested that fluctuating goal maintenance mechanisms that eventually result in a correct response could be observed independently of stronger failures in goal maintenance in which participants are effectively producing errors. It is therefore plausible that the goal primes used in the present study were not a strong enough facilitator of goal maintenance to influence the more extreme form of goal neglect and thus that one interprets the present results as showing that facilitation is unaffected by the primes. However, this latter position fails to account for the increase in RTs to congruent trials. In sum, the present results are best interpreted as showing that the goal primes facilitate the colour naming goal and as a consequence renders less efficient the processes of visual word recognition which slows colour classification to neutral and congruent trials, but speeds up responses to incongruent trials.

An alternative explanation of the present results is that the goal primes serve as positive, rewarding stimuli, because they are consistent with the task goal, thereby inducing an emotional response that somehow aids performance on the Stroop task. Indeed previous research has shown that primes producing positive affect can modulate Stroop task performance. Kuhl and Kazen [23] have shown that words connoting positive emotions (e.g. hero, friend, pleasure, happiness) presented for 250 ms, 700 ms, or 1000 ms prior to the onset of a Stroop stimulus can eliminate Stroop interference, whereas words connoting negative emotions have no effect on performance. However, there are good reasons to believe that these priming effects are not operating via the same mechanism. The effect observed by Kuhl and Kazen [23] was observed only when two consecutive Stroop trials followed the presentation of the prime. When only one Stroop trial was presented immediately following the prime words (as in the present study), there was no observed effect on performance. Therefore, unlike the present results the effect of emotion prime words on performance was dependent on the participants preparing to perform two Stroop trials in succession. Kuhl and Kazen interpreted this requirement for two consecutive Stroop trials as the result of positive primes acting specifically on the sequencing of action steps needed when the task requires participants to perform two Stroop trials in a row. Thus, the effect observed by Kuhl and Kazen and the effect presented here seem to operate via different mechanisms.

In the context of the Stroop task, the role of intentional executive control is to ensure that the colour naming goal is activated enough to override the effect of obligatorily processing the irrelevant word. Since word reading proceeds despite the intention to ignore the irrelevant word, executive control needs to exert enough control to supress the word reading process and the consequent evidence towards an incorrect response. The robust nature of the Stroop effect suggests that executive control usually fails to exert sufficient control. The somewhat ironic effect presented here suggests that the very process that should be suppressed actually aids executive control by ensuring that the task goal is more activated than it would otherwise be. The obligatory reading of the prime appears to enhance the control mechanisms that exert control over goal representations. Therefore, the present results support those from a recent study showing that executive control can be influenced by automatic processing [24] and show that exogenously activated concepts consistent with an intentionally activated goal can facilitate goal-oriented behaviour.
